# Magnetized and non-magnetized Casson fluid flow with gyrotactic microorganisms over a stratified stretching cylinder

**DOI:** 10.1038/s41598-021-95878-8

**Published:** 2021-08-12

**Authors:** Abdullah Dawar, Zahir Shah, Hashim M. Alshehri, Saeed Islam, Poom Kumam

**Affiliations:** 1 Department of Mathematics, Abdul Wali Khan University, Mardan, Mardan, 23200 Khyber Pakhtunkhwa Pakistan; 2 Department of Mathematical Sciences, University of Lakki Marwat, Lakki Marwat, 28420 Khyber Pakhtunkhwa Pakistan; 3 Department of Mathematics, Faculty of Science, King Abdulaziz University, Jeddah, 21521 Saudi Arabia; 4 Fixed Point Research Laboratory, Fixed Point Theory and Applications Research Group, Center of Excellence in Theoretical and Computational Science (TaCS-CoE), Faculty of Science, King Mongkut’s University of Technology Thonburi (KMUTT), 126 Pracha Uthit Rd., Bang Mod, Thung Khru, Bangkok, 10140 Thailand; 5 Department of Medical Research, China Medical University Hospital, China Medical University, Taichung, 40402 Taiwan

**Keywords:** Applied mathematics, Mechanical engineering

## Abstract

This study presents the magnetized and non-magnetized Casson fluid flow with gyrotactic microorganisms over a stratified stretching cylinder. The mathematical modeling is presented in the form of partial differential equations and then transformed into ordinary differential equations (ODEs) utilizing suitable similarity transformations. The analytical solution of the transformed ODEs is presented with the help of homotopy analysis method (HAM). The convergence analysis of HAM is also presented by mean of figure. The present analysis consists of five phases. In the first four phases, we have compared our work with previously published investigations while phase five is consists of our new results. The influences of dimensionless factors like a magnetic parameter, thermal radiation, curvature parameter, Prandtl number, Brownian motion parameter, Schmidt number, heat generation, chemical reaction parameter, thermophoresis parameter, Eckert number, and concentration difference parameter on physical quantities of interests and flow profiles are shown through tables and figures. It has been established that with the increasing Casson parameter (i.e. $\beta \to \infty$), the streamlines become denser which results the increasing behavior in the fluid velocity while on the other hand, the fluid velocity reduces for the existence of Casson parameter (i.e. $\beta = 1.0$). Also, the streamlines of stagnation point Casson fluid flow are highly wider for the case of magnetized fluid as equated to non-magnetized fluid. The higher values of bioconvection Lewis number, Peclet number, and microorganisms’ concentration difference parameter reduces the motile density function of microorganisms while an opposite behavior is depicted against density number.

## Introduction

Magnetohydrodynamic (MHD) flow is a fluid flow that interacts with an applied magnetic field. Newtonian and non-Newtonian versions are used to engage the fluid. Commonly well-known Newtonian fluids are gasoline, alcohol, water, and minerals. When it comes to the study of MHD, the inferred fluid model will serve as the electrical conductor. To better understand the significance of MHD flows, scientists suggested linking the integrated magnetic field with Navier–Stokes equations, and the final results identify appropriate remarks in both engineering and industrial areas such as electrolytic Hall cells, plasma welding, casting processes, defensive eccentricities, and thermal transmission characteristics are some examples. Numerous researchers and scientists expressed their perspectives on the role of magnetic field participation like Walker and Hua^1^ discussed the interaction of magnetic field over rectangular ducts. Chaturvedi^2^ addressed the MHD flow of viscous fluid with variable suction through a porous plate. Aldoss^3^ used a vertical cylinder to investigate the influence of magnetic field using the non-Darcian model. He discovered that when a magnetic field is introduced, both normal and forced convection regimes produce a reverse effect. Nanousis^4^ signified the time-dependent MHD flow of viscous fluid though an oscillatory surface. The MHD flow of electrically conducting fluid over a thermally stratified medium was addressed by Chamkha^5^. The heat transfer in MHD stagnation point flow of micropolar fluid over a three-dimensional frame was addressed by Gupta and Bhattacharyya^6^. The micropolar fluid flow with Joule heating and magnetic impacts is represented by Hakiem et al.^7^. The mass and heat transfer characteristics of MHD micropolar fluid flow through a circular cylinder was analyzed by Mansour et al.^8^. The thermal transmission characteristics of MHD fluid flow in the existence of thermal radiation were proposed by Sadeek^9^. The flow of micropolar fluid with MHD and constant suction impacts was presented by Amin^10^. The MHD flow of Oldroyd-B fluid was addressed by Hayat et al.^11^. The MHD flow of Casson fluid through a shrinking surface was discussed by Nadeem et al.^12^. Thammanna et al.^13^ addressed the chemically reactive MHD flow of Casson fluid through an unsteady stretching sheet with convective boundary conditions. Ramesh et al.^14^ presented the MHD Casson fluid flow with Cattaneo–Christov heat theory heat absorption/generation. The MHD flow of nanofluid past an unsteady contracting cylinder with convective condition and heat generation/absorption was investigated by Ramesh et al.^15^. Bilal et al.^16^ presented the numerical analysis of MHD viscoelastic fluid past an exponentially extending sheet. The related studies towards this development can be seen in Refs.^17–46^.

Because of its useful applications in engineering and industry, a study of stratification phenomena in non-Newtonian fluids has got a lot of interest. Temperature fluctuations, composition variations, or a combination of different liquids of varying thicknesses causes stratification of the medium. For instance, thermal rejection into the atmosphere, storage systems of heat energy, geophysical flows, etc. In short, stratification takes place in both natural and industrial phenomena. Using thermal and solutal stratifications many attempts have been performed like Yang et al.^47^ analyzed the convective fluid flow over a thermally stratified medium. The buoyance flow over a stratified medium was discussed by Jaluria and Gebhart^48^. The mixed convection fluid flow over a stratified medium was introduced by Ishak et al.^49^. The thermally stratified flow of micropolar fluid over a vertical plate constant and uniform heat flux was examined by Chang and Lee^50^. The incompressible and electrically conducting viscous fluid flow through an inclined plate was presented by Singh and Makinde^51^. The MHD flow of dissipative fluid with heat generation and second-order chemical reaction was introduced by Malik and Rehman^52^. The incompressible and mixed convective flow of viscous fluid through a thermally stratified stretching cylinder was introduced by Mukhopadhyay and Ishak^53^. Cheng et al.^54^ investigated the mass and heat transmission in a power-law fluid through a stratified medium. The heat transmission in a boundary layer flow through a stratified vertical plate was presented by Ibrahim and Makinde^55^. Furthermore, related analyses are mentioned in Refs.^56–60^.

Our contribution to the field of non-Newtonian fluids consists of Casson fluid flow containing gyrotactic microorganisms through a stratified stretching cylinder. Furthermore, stagnation point, Joule heating, heat absorption/generation, thermal stratification, mass stratification, motile stratification, nonlinear thermal radiation, magnetic field, and chemical reaction are taken into account. Also, the fluid flow is treated for magnetized and non-magnetized conditions. The present analysis consists of five phases. In the first four phases, we have compared our work with previously published investigations while phase five is consists of our new results.

At the end of this analysis, we will be able to answer that:

- How the streamlines behave for non-Newtonian (Casson) and Newtonian fluid?
- How the streamlines behave for magnetized and non-magnetized Casson fluid flow under the stagnation point?
- What are the impacts of bioconvection Lewis number, Peclet number, and microorganisms’ concentration difference parameter on Casson fluid flow?
- What are the impacts of bioconvection Lewis number, Peclet number, and microorganisms’ concentration difference parameter on density number?

## Problem formulation

The mathematical model for Casson fluid containing gyrotactic microorganisms through a stretching cylinder is modeled under the effects of various parameters like stagnation point, Joule heating, heat absorption/generation, thermal stratification, mass stratification, motile stratification, thermal radiation, magnetic field, and chemical reaction.

For an isotropic and incompressible flow of Casson fluid, the rheological equation is stated as^61^:

$$ \tau_{ij} = \left\{ {\begin{array}{*{20}l} {2\left( {\mu_{B} + \frac{{p_{y} }}{{\sqrt {2\pi } }}} \right)e_{ij} ,\,\,\,\pi > \pi_{c} } \hfill \\ {2\left( {\mu_{B} + \frac{{p_{y} }}{{\sqrt {2\pi_{c} } }}} \right)e_{ij} ,\,\,\,\pi < \pi_{c} } \hfill \\ \end{array} } \right., $$

where $\tau_{ij}$ is the $\left( {i,j} \right){\text{th}}$ component of stress tensor, $\pi = e_{ij} e_{ij}$ and $e_{ij}$ are the $\left( {i,j} \right){\text{th}}$ component of the deformation rate, $p_{y}$ is the fluid yield stress, and $\mu_{B}$ is the plastic dynamic viscosity of the non-Newtonian fluid, $\pi$ is the product of component of deformation rate with itself and $\pi_{c}$ is the critical value of this product.

According to these assumptions the leading equations are^38,57,60^:

1 $$ \frac{{\partial \left( {rU} \right)}}{\partial x} + \frac{{\partial \left( {rV} \right)}}{\partial r} = 0, $$

2 $$ U\frac{\partial U}{{\partial x}} + V\frac{\partial V}{{\partial r}} = U_{e} \frac{{\partial U_{e} }}{\partial x} + \nu \left( {1 + \frac{1}{\beta }} \right)\left( {\frac{1}{r}\frac{\partial U}{{\partial r}} + \frac{{\partial^{2} U}}{{\partial r^{2} }}} \right) - \frac{{\sigma B_{0}^{2} }}{\rho }\left( {U - U_{e} } \right), $$

3 $$ \begin{aligned} U\frac{\partial T}{{\partial x}} + V\frac{\partial T}{{\partial r}} & = \frac{\alpha }{r}\frac{\partial }{\partial r}\left( {r\frac{\partial T}{{\partial r}}} \right) + \frac{1}{{\rho c_{p} }}\frac{1}{r}\frac{{4\sigma^{*} }}{{3k^{*} }}\frac{\partial }{\partial r}\left( {r\frac{{\partial T^{4} }}{\partial r}} \right) \\ & \quad + \tau \left( {D_{B} \frac{\partial T}{{\partial r}}\frac{\partial C}{{\partial r}} + \frac{{D_{T} }}{{T_{\infty } }}\left( {\frac{\partial T}{{\partial r}}} \right)^{2} } \right) + \frac{{Q_{0} }}{{\rho c_{p} }}\left( {T - T_{\infty } } \right) + \frac{{\sigma B_{0}^{2} }}{{\rho c_{p} }}U^{2} , \\ \end{aligned} $$

4 $$ U\frac{\partial C}{{\partial x}} + V\frac{\partial C}{{\partial r}} = D_{B} \left( {\frac{1}{r}\frac{\partial C}{{\partial r}} + \frac{{\partial^{2} C}}{{\partial r^{2} }}} \right) + \frac{{D_{T} }}{{T_{\infty } }}\left( {\frac{1}{r}\frac{\partial T}{{\partial r}} + \frac{{\partial^{2} T}}{{\partial r^{2} }}} \right) - R_{0} \left( {C - C_{\infty } } \right), $$

5 $$ U\frac{\partial N}{{\partial x}} + V\frac{\partial N}{{\partial r}} = D_{n} \left( {\frac{1}{r}\frac{\partial N}{{\partial r}} + \frac{{\partial^{2} N}}{{\partial r^{2} }}} \right) - \frac{{\alpha W_{c} }}{{C_{w} - C_{\infty } }}\left( {\frac{\partial N}{{\partial r}}\frac{\partial C}{{\partial r}} + N\frac{{\partial^{2} C}}{{\partial r^{2} }}} \right), $$

with boundary conditions^57^:

6 $$ \left. {\begin{array}{*{20}l} {U = \overline{U}\left( x \right) = ax,\,\,\,V = 0,\,\,\,T\left( {x,r} \right) = T_{w} \left( x \right) = T_{0} + {{bx} \mathord{\left/ {\vphantom {{bx} L}} \right. \kern-\nulldelimiterspace} L},\,\,\,C\left( {x,r} \right) = C_{w} \left( x \right) = C_{0} + {{dx} \mathord{\left/ {\vphantom {{dx} L}} \right. \kern-\nulldelimiterspace} L},\,\,\,} \hfill \\ {N\left( {x,r} \right) = N_{w} \left( x \right) = N_{0} + {{gx} \mathord{\left/ {\vphantom {{gx} L}} \right. \kern-\nulldelimiterspace} L}\,\,\,\,at\,\,\,\,r = R,} \hfill \\ {U \to U_{e} = a^{*} x,\,\,\,T\left( {x,r} \right) \to T_{\infty } \left( x \right) = T_{0} + {{cx} \mathord{\left/ {\vphantom {{cx} L}} \right. \kern-\nulldelimiterspace} L},\,\,\,C\left( {x,r} \right) \to C_{\infty } \left( x \right) = C_{0} + {{ex} \mathord{\left/ {\vphantom {{ex} L}} \right. \kern-\nulldelimiterspace} L},\,\,\,} \hfill \\ {N\left( {x,r} \right) \to N_{\infty } \left( x \right) = N_{0} + {{hx} \mathord{\left/ {\vphantom {{hx} L}} \right. \kern-\nulldelimiterspace} L}\,\,\,as\,\,\,r \to \infty } \hfill \\ \end{array} } \right\}, $$

The conversion parameters are defined as:

7 $$ \begin{aligned} & \psi = R\sqrt {\frac{{\nu U_{0} x^{2} }}{L}} f\left( \eta \right),\,\,\,U = \frac{1}{r}\frac{\partial \psi }{{\partial r}} = \frac{{U_{0} x}}{L}f^{\prime}\left( \eta \right),\,\,\,V = - \frac{1}{r}\frac{\partial \psi }{{\partial x}} = - \frac{R}{r}\sqrt {\frac{{\nu U_{0} }}{L}} f\left( \eta \right), \\ & \eta = \frac{{r^{2} - R^{2} }}{2R}\sqrt {\frac{{U_{0} }}{\nu L}} ,\,\,\,\theta \left( \eta \right) = \frac{{T - T_{\infty } }}{{T_{w} - T_{0} }},\,\,\,\phi \left( \eta \right) = \frac{{C - C_{\infty } }}{{C_{w} - C_{0} }},\,\,\,\chi \left( \eta \right) = \frac{{N - N_{\infty } }}{{N_{w} - N_{0} }}. \\ \end{aligned} $$

Using (7), (2–6) are reduced as:

8 $$ \frac{1}{\beta }\left( {1 + 2\kappa \xi } \right)f^{\prime\prime\prime} + \frac{2}{\beta }\kappa f^{\prime\prime} + \left( {1 + 2\kappa \xi } \right)f^{\prime\prime\prime} + 2\kappa f^{\prime\prime} + ff^{\prime\prime} - f^{{\prime}{2}} - \gamma^{2} \left( {f^{\prime} - A} \right) + A^{2} = 0, $$

9 $$ \begin{aligned} & \left( {1 + 2\kappa \xi } \right)\left( {1 + \frac{4}{3}Rd} \right)\theta^{\prime\prime} + 2\kappa \left( {1 + \frac{4}{3}Rd} \right)\theta^{\prime} + \Pr {\text{Nb}}\left( {1 + 2\kappa \xi } \right)\left( {\theta^{\prime}\phi^{\prime}} \right) \\ & + \Pr \left( {f\theta^{\prime} - \delta_{1} f^{\prime} + Q\theta - \theta f^{\prime} + {\text{Ec}}\gamma^{2} f^{2} } \right) + \Pr {\text{Nb}}\left( {1 + 2\kappa \xi } \right)\left( {\frac{{{\text{Nt}}}}{{{\text{Nb}}}}\theta^{{\prime}{2}} } \right) = 0, \\ \end{aligned} $$

10 $$ \left( {1 + 2\kappa \xi } \right)\left[ {\phi^{\prime\prime} + \frac{{{\text{Nt}}}}{{{\text{Nb}}}}\theta^{\prime\prime}} \right] + Sc\left( {f\phi^{\prime} - \delta_{2} f^{\prime} - \phi f^{\prime}} \right) + 2\kappa \left( {\frac{{{\text{Nt}}}}{{{\text{Nb}}}}\theta^{\prime} + \phi^{\prime}} \right) - R_{c} \phi = 0, $$

11 $$ \begin{aligned} & \left( {1 + 2\kappa \xi } \right)\chi^{\prime\prime} + 2\kappa \chi^{\prime} - {\text{Le}}\left( {f^{\prime}\chi - f\chi^{\prime} + S_{3} f^{\prime}} \right) \\ & - {\text{Pe}}\left( {1 + 2\kappa \xi } \right)\phi^{\prime}\chi^{\prime} - 2\kappa {\text{Pe}}\left[ {\Omega + \chi } \right]\phi^{\prime} - {\text{Pe}}\left( {1 + 2\kappa \xi } \right)\left[ {\Omega + \chi } \right]\phi^{\prime\prime} = 0, \\ \end{aligned} $$

with transformed boundary conditions:

12 $$ \left. {\begin{array}{*{20}l} {f^{\prime}\left( \xi \right) = 1,\,\,\,f\left( \xi \right) = 0,\,\,\,\theta \left( \xi \right) = 1 - \delta_{1} ,\,\,\,\phi \left( \xi \right) = 1 - \delta_{2} ,\,\,\,\chi \left( \xi \right) = 1 - \delta_{3} \,\,\,at\,\,\,\xi = 0,} \hfill \\ {f^{\prime}\left( \xi \right) \to A,\,\,\,\theta \left( \xi \right) \to 0,\,\,\,\phi \left( \xi \right) \to 0,\,\,\,\chi \left( \xi \right) \to 0\,\,\,as\,\,\,\xi \to \infty } \hfill \\ \end{array} } \right\}. $$

where $\kappa = \frac{1}{R}\sqrt {\frac{\nu L}{{U_{0} }}}$ is the curvature parameter,$\gamma = \sqrt {\frac{{\sigma B_{0}^{2} }}{a\rho }}$ is the magnetic parameter,$A = \frac{{a^{*} }}{a}$ is the ratio of velocities, $Rd = \frac{{4\sigma^{*} T_{\infty }^{3} }}{{kk^{*} }}$ is the thermal radiation parameter, $\Pr = \frac{\nu }{\alpha }$ is the Prandtl number,${\text{Nb}} = \frac{{\tau D_{B} \left( {C_{w} - C_{\infty } } \right)}}{\nu }$ is the Brownian motion parameter,${\text{Nt}} = \frac{{\tau D_{T} \left( {T_{w} - T_{\infty } } \right)}}{{\nu T_{\infty } }}$ is the thermophoresis parameter,$\delta_{1} = \frac{c}{b}$ is the temperature stratification parameter,$\delta_{2} = \frac{e}{d}$ is the concentration stratification parameter, $\delta_{3} = \frac{h}{g}$ is the motile density stratification parameter,$Q = \frac{{Q_{0} L}}{{\rho c_{p} U_{0} }}$ is the heat generation parameter,${\text{Sc}} = \frac{\nu }{{D_{B} }}$ is the Schmidt number,$R_{c} = \frac{{LR_{0} }}{{U_{0} }}$ is the chemical reaction parameter, ${\text{Le}} = \frac{\alpha }{{D_{n} }}$ is bioconvection Lewis number, and ${\text{Ec}} = \frac{{xU_{0} }}{{Lc_{p} \left( {T_{w} - T_{\infty } } \right)}}$ is Eckert number, ${\text{Pe}} = \frac{{bW_{c} }}{{D_{n} }}$ is the bioconvection Peclet number, $\Omega = \frac{{N_{\infty } }}{{N_{\infty } - N_{0} }}$ is the concentration difference parameter.

## Engineering quantities of interests

### Skin friction (SF)

At the cylindrical surface, the SF is specified by:

13 $$ C_{f} = \frac{{\tau_{w} }}{{\rho \frac{{U^{2} }}{2}}} = \frac{{\mu \left( {1 + \frac{1}{\beta }} \right)\left. {\frac{\partial u}{{\partial r}}} \right|_{r = R} }}{{\rho \frac{{U^{2} }}{2}}}, $$

In view of Eq. (7), the dimensionless form of SF is written as:

14 $$ \frac{1}{2}{\text{Re}}_{x}^{\frac{1}{2}} C_{f} = \left( {1 + \frac{1}{\beta }} \right)f^{\prime\prime}\left( 0 \right), $$

where ${\text{Re}}_{x} = \frac{{U_{0} x^{2} }}{\nu L}$ is the local Reynolds number.

### Nusselt number (NN)

At the surface of cylinder, the NN is given by:

15 $$ {\text{Nu}}_{x} = \frac{{xq_{w} }}{{k\left( {T_{w} - T_{0} } \right)}} = \frac{{ - k\left. {\frac{\partial T}{{\partial r}}} \right|_{r = R} }}{{k\left( {T_{w} - T_{0} } \right)}} - \frac{4}{3}\frac{{\sigma^{*} }}{{k^{*} }}\left. {\frac{{\partial T^{4} }}{\partial r}} \right|_{r = R} , $$

In view of Eq. (7), the dimensionless form of NN is written as:

16 $$ {\text{Re}}_{x}^{{ - \frac{1}{2}}} {\text{Nu}}_{x} = - \left( {1 + \frac{4}{3}Rd} \right)\theta^{\prime}\left( 0 \right). $$

### Sherwood number (SN)

At the surface of cylinder, the SN is given by:

17 $$ {\text{Sh}}_{x} = \frac{{xj_{w} }}{{D\left( {C_{w} - C_{0} } \right)}} = \frac{{ - D\left. {\frac{\partial C}{{\partial r}}} \right|_{r = R} }}{{D\left( {C_{w} - C_{0} } \right)}}, $$

In view of Eq. (7), the dimensionless form of SN is written as:

18 $$ {\text{Re}}_{x}^{{ - \frac{1}{2}}} {\text{Sh}}_{x} = - \phi^{\prime}\left( 0 \right). $$

### Density number (DN)

At the surface of cylinder, the DN is given by:

19 $$ Dn_{x} = \frac{{xq_{n} }}{{D_{n} \left( {N_{w} - N_{0} } \right)}} = \frac{{ - D_{n} \left. {\frac{\partial N}{{\partial r}}} \right|_{r = R} }}{{D_{n} \left( {N_{w} - N_{0} } \right)}}, $$

In view of Eq. (7), the dimensionless form of SN is written as:

20 $$ {\text{Re}}_{x}^{{ - \frac{1}{2}}} Dn_{x} = - \chi^{\prime}\left( 0 \right). $$

## HAM solution and convergence

An analytical of the modeled problem is solved with the help of HAM. The initial guesses and linear operators are taken as:

21 $$ L_{f} \left( f \right) = f^{\prime\prime\prime} - f^{\prime},\,\,\,L_{\theta } \left( \theta \right) = \theta^{\prime\prime} - \theta ,\,\,\,L_{\phi } \left( \phi \right) = \phi^{\prime\prime} - \phi ,\,\,\,L_{\chi } \left( \chi \right) = \chi^{\prime\prime} - \chi , $$

22 $$ f_{0} \left( \xi \right) = \xi + A\xi - A\xi e^{ - \xi } ,\,\,\,\theta_{0} \left( \xi \right) = \left( {1 - \delta_{1} } \right)e^{ - \xi } ,\,\,\,\phi_{0} \left( \xi \right) = \left( {1 - \delta_{2} } \right)e^{ - \xi } ,\,\,\,\chi_{0} \left( \xi \right) = \left( {1 - \delta_{3} } \right)e^{ - \xi } , $$

with23where  are the arbitrary constants.

**Figure 1:**
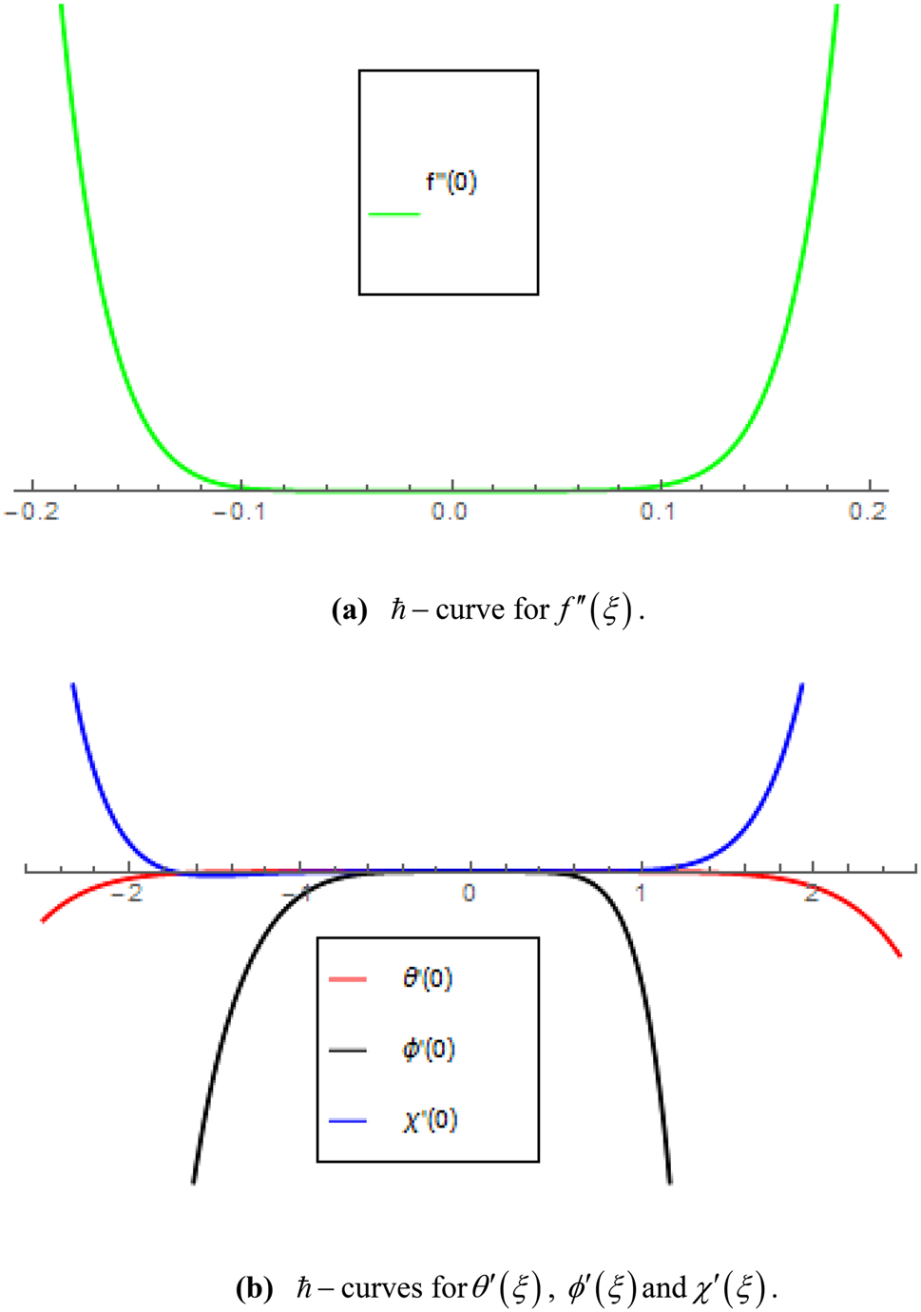
(**a**) $\hbar$-curve for $f^{\prime\prime}\left( \xi \right)$. (**b**) $\hbar$-curves for $\theta^{\prime}\left( \xi \right)$, $\phi^{\prime}\left( \xi \right)$ and $\chi^{\prime}\left( \xi \right)$.

The convergence analysis of HAM is presented in Fig. 1a,b. The convergence area for $f^{\prime\prime}\left( 0 \right)$ is $- 0.1 \le \hbar_{f} \le 0.1$ as shown in Fig. 1a. Similarly, the convergence areas for $\theta^{\prime}\left( 0 \right)$, $\phi^{\prime}\left( 0 \right)$ and $\chi^{\prime}\left( 0 \right)$ are $- 1.75 \le \hbar_{\theta } \le 1.50$, $- 0.75 \le \hbar_{\phi } \le 1.60$ and $- 1.75 \le \hbar_{\chi } \le 1.20$ respectively, as shown in Fig. 1b.

## Phases of study

This paper contains the generalized mathematical formulation for both magnetized and non-magnetized Casson fluid flow with gyrotactic microorganisms over a stratified cylinder. So the present analysis divides the proposed study into sub-phases in order to analyze the Casson fluid flow under different situations. It should be noted that in the current investigation, we have ignored the mixed convection phenomena. The present analysis consists of five phases. In the first four phases, we have compared our work with previously published investigations while phase five is consists of our new results.

### Phase 1

Ignoring the suspended nanoparticles assumptions, stagnation point, Joule heating, thermal radiation, concentration distribution, and motile distribution the comprehensive formulation compact to the problem described in Ref.^38^. In this study, the comparative analysis of MHD mixed convective flow of Casson fluid through the stratified flat and cylindrical stretching surfaces are analyzed. The obtained variations were found to be surprisingly large for cylindrical geometry as particularly in comparison to plane surface geometry. The SF is found the heightening function of $\kappa$,$\Pr$,$\beta$ and $\delta_{1}$ whereas a reducing function of $Q$. The NN is found the heightening function of $\kappa$ and $\Pr$ whereas a diminishing function of $Q$,$\delta_{1}$ and $\beta$. By applying the assumptions reported in Ref.^38^, an analytical solution is proposed in order to verify the current model. An outstanding comparison is found with Ref.^38^ as shown in Table 1. The mathematical formulation designed in Ref.^38^ is given as:

24 $$ \frac{1}{\beta }\left( {1 + 2\kappa \xi } \right)f^{\prime\prime\prime} + \frac{1}{\beta }2\kappa f^{\prime\prime} + \left( {1 + 2\kappa \xi } \right)f^{\prime\prime\prime} + 2\kappa f^{\prime\prime} + ff^{\prime\prime} - f^{{\prime}{2}} - \gamma^{2} f^{\prime} = 0, $$

25 $$ \left( {1 + 2\kappa \xi } \right)\theta^{\prime\prime} + 2\kappa \theta^{\prime} + \Pr \left( {f\theta^{\prime} - \delta_{1} f^{\prime} + Q\theta - \theta f^{\prime}} \right) = 0, $$

**Table 1:** Comparison of SF and NN with Ref.^38^.

$K$	$\beta$	$\Pr$	$\delta_{1}$	$Q^{*}$	$0.5C_{f} {\text{Re}}_{x}^{{{1 \mathord{\left/ {\vphantom {1 2}} \right. \kern-\nulldelimiterspace} 2}}}$		${\text{Re}}_{x}^{{ - {1 \mathord{\left/ {\vphantom {1 2}} \right. \kern-\nulldelimiterspace} 2}}} {\text{Nu}}_{x}$	
					Ref.^38^	Present results	Ref.^38^	Present results
0.1		0.1	0.1	0.1	− 0.3826	− 0.384657	0.8254	0.827743
0.2					− 0.4036	− 0.407543	0.8599	0.856536
0.3					− 0.4248	− 0.427654	0.8948	0.897864
0.1	1.3				− 0.3826	− 0.389764	0.8254	0.827743
	1.4				− 0.3884	− 0.389532	0.8225	0.827532
	1.5				− 0.3928	− 0.396531	0.8200	0.820026
		0.6			− 0.3816	− 0.385424	0.7542	0.758086
		0.7			− 0.3826	− 0.384657	0.8254	0.827743
		0.8			− 0.3835	− 0.387532	0.8930	0.893707
		0.1	0.1		− 0.3826	− 0.384657	0.8254	0.827743
			0.2		− 0.3851	− 0.387543	0.7958	0.790862
			0.3		− 0.3877	− 0.388932	0.7662	0.769741
			0.1	0.1	− 0.3826	− 0.384657	0.8254	0.827743
				0.2	− 0.3821	− 0.389743	0.7789	0.779987
				0.3	− 0.3813	− 0.389851	0.7225	0.724901

with transformed boundary conditions:

26 $$ \left. {\begin{array}{*{20}l} {f\left( \xi \right) = 0,\,\,\,f^{\prime}\left( \xi \right) = 1,\,\,\,\theta \left( \xi \right) = 1 - \delta_{1} \,\,at\,\,\,\xi = 0,} \hfill \\ {f^{\prime}\left( \xi \right) \to 0,\,\,\,\theta \left( \xi \right) \to 0,\,\,\,as\,\,\,\xi \to \infty } \hfill \\ \end{array} } \right\}. $$

### Phase 2

In the absence of suspended nanoparticles assumptions, Joule heating, thermal radiation, stagnation point, and motile distribution the comprehensive formulation compact to the problem described in Ref.^56^. In this analysis, a stratified flow of Casson fluid through an inclined cylinder was presented. It was concluded that the SF is reduced with $K$,$\beta$,$\Pr$ and $\delta_{1}$ whereas the opposite trend in SF was found due to $\delta_{2}$ and $Q^{*}$. Furthermore, the NN has reduced with $\beta$,$\delta_{1}$,$Q^{*}$ however the NN has increased with $K$ and $\Pr$. Additionally, it was also concluded that the higher $K$,$\delta_{2}$,$R_{c}$ and $Sc$ have increasing impacts on SN. By applying the assumptions reported in Ref.^56^, an analytical solution is proposed in order to verify the current model. An outstanding comparison is found with Ref.^56^ as shown in Tables 2 and 3. The mathematical formulation designed in Ref.^56^ is given as:

27 $$ \left( {1 + 2\kappa \xi } \right)f^{\prime\prime\prime} + 2\kappa f^{\prime\prime} + \frac{1}{\beta }\left[ {\left( {1 + 2\kappa \xi } \right)f^{\prime\prime\prime} + 2\kappa f^{\prime\prime}} \right] + ff^{\prime\prime} - f^{{\prime}{2}} - \gamma^{2} f^{\prime} = 0, $$

28 $$ \left( {1 + 2\kappa \xi } \right)\theta^{\prime\prime} + 2\kappa \theta^{\prime} + \Pr \left( {f\theta^{\prime} - \delta_{1} f^{\prime} + Q\theta - \theta f^{\prime}} \right) = 0, $$

29 $$ \left( {1 + 2\kappa \xi } \right)\phi^{\prime\prime} + {\text{Sc}}\left( {f\phi^{\prime} - \delta_{2} f^{\prime} - \phi f^{\prime} - R_{c} \phi } \right) = 0, $$

**Table 2:** Comparison of SF and NN with Ref.^56^.

$K$	$\beta$	$\Pr$	$Q^{*}$	$\delta_{1}$	$\delta_{2}$	${\text{Sc}}$	$0.5C_{f} {\text{Re}}_{x}^{{{1 \mathord{\left/ {\vphantom {1 2}} \right. \kern-\nulldelimiterspace} 2}}}$		${\text{Re}}_{x}^{{ - {1 \mathord{\left/ {\vphantom {1 2}} \right. \kern-\nulldelimiterspace} 2}}} {\text{Nu}}_{x}$	
							Ref.^56^	Present results	Ref.^56^	Present results
0.2	0.1	0.1	0.1	0.1	0.1	0.1	− 2.1020	− 2.105643	0.4260	0.425475
0.4							− 3.0094	− 3.008684	0.5331	0.538864
0.6							− 4.0514	− 4.057905	0.6326	0.637532
0.1	1.1						− 1.7590	− 1.753242	0.3667	0.365416
	1.2						− 1.7840	− 1.789031	0.3663	0.366904
	1.3						− 1.8066	− 1.807863	0.3659	0.365732
		0.3					− 1.8667	− 1.866943	0.4223	0.429654
		0.4					− 1.8690	− 1.866584	0.4507	0.456895
		0.5					− 1.8711	− 1.876507	0.5091	0.503683
		0.1	0.2				− 1.7304	− 1.735822	0.3947	0.398426
			0.4				− 1.7300	− 1.730482	0.3810	0.387953
			0.6				− 1.7292	− 1.726943	0.3671	0.369754
			0.1	0.2			− 1.7288	− 1.726089	0.4059	0.408063
				0.4			− 1.7456	− 1.742018	0.4030	0.403573
				0.6			− 1.7626	− 1.761069	0.4003	0.406842
				0.1	0.2		− 1.7332	− 1.737857	–	–
					0.4		− 1.7228	− 1.725992	–	–
					0.6		− 1.7202	− 1.727042	–	–
					0.1	0.2	− 1.7422	− 1.742917	–	0.506483
						0.4	− 1.7312	− 1.730793	–	0.657432
						0.6	− 1.7316	− 1.733025	–	0.805824

**Table 3:** Comparison of SN with Ref.^56^.

$K$	Sc	$\delta_{2}$	$R_{c}$	${\text{Re}}_{x}^{{ - {1 \mathord{\left/ {\vphantom {1 2}} \right. \kern-\nulldelimiterspace} 2}}} {\text{Sh}}_{x}$	
				Ref.^56^	Present results
0.2	0.1	0.1	0.1	0.4702	0.470536
0.3				0.5107	0.510976
0.4				0.5725	0.575679
0.1	0.2			0.4860	0.484784
	0.3			0.5544	0.557743
	0.4			0.6202	0.628537
	0.1	0.2		0.2434	0.248954
		0.3		0.3324	0.338736
		0.4		0.4215	0.428636
		0.1	0.2	0.4213	0.428535
			0.3	0.4343	0.438080
			0.4	0.4470	0.448835

with transformed boundary conditions:

30 $$ \left. {\begin{array}{*{20}l} {f^{\prime}\left( \xi \right) = 1,\,\,\,f\left( \xi \right) = 0,\,\,\,\theta \left( \xi \right) = 1 - \delta_{1} ,\,\,\,\phi \left( \xi \right) = 1 - \delta_{2} \,\,at\,\,\,\xi = 0,} \hfill \\ {f^{\prime}\left( \xi \right) \to 0,\,\,\,\theta \left( \xi \right) \to 0,\,\,\,\phi \left( \xi \right) \to 0,\,\,\,as\,\,\,\xi \to \infty } \hfill \\ \end{array} } \right\}. $$

### Phase 3

In the absence of suspended nanoparticles assumptions, Joule heating, thermal radiation, stagnation point, magnetic field, concentration distribution, motile distribution, and with the condition when $\beta \to \infty$, the comprehensive formulation condensed to the problem reported in Ref.^53^. It was found that the buoyancy force escalates the SF and the thermal stratification parameter increases the NN. In addition, it was claimed that the SF and NN are highly impressed for a cylinder in contrast with plate. The mathematical formulation designed in Ref.^53^ is given as:

31 $$ \left( {1 + 2\kappa \xi } \right)f^{\prime\prime\prime} + 2\kappa f^{\prime\prime} + ff^{\prime\prime} - f^{{\prime}{2}} = 0, $$

32 $$ \left( {1 + 2\kappa \xi } \right)\theta^{\prime\prime} + 2\kappa \theta^{\prime} + \Pr \left( {f\theta^{\prime} - \delta_{1} f^{\prime} - \theta f^{\prime}} \right) = 0, $$

with transformed boundary conditions:

33 $$ \left. {\begin{array}{*{20}l} {f\left( \xi \right) = 0,\,\,\,f^{\prime}\left( \xi \right) = 1,\,\,\,\theta \left( \xi \right) = 1 - \delta_{1} \,\,\,at\,\,\,\xi = 0,} \hfill \\ {f^{\prime}\left( \xi \right) \to 0,\,\,\,\theta \left( \xi \right) \to 0,\,\,\,as\,\,\,\xi \to \infty } \hfill \\ \end{array} } \right\}. $$

### Phase 4

By ignoring the Joule heating and motile distribution, the comprehensive formulation condensed to the problem reported in Ref.^57^. In this examination, the authors found that the SF reduces with $K$ and $\beta$. Furthermore, the NN increases with $K$ and $\Pr$ whereas as reduces with $\delta_{1}$. Also, the SN reduces with $K$,$\delta_{2}$ and increases with ${\text{Le}}$ and $\Pr$. An outstanding comparison is found with Ref.^57^ as shown in Tables 4, 5 and 6. The mathematical formulation designed in Ref.^57^ is given as:

34 $$ \frac{1}{\beta }\left( {1 + 2\kappa \xi } \right)f^{\prime\prime\prime} + \frac{1}{\beta }2\kappa f^{\prime\prime} + \left( {1 + 2\kappa \xi } \right)f^{\prime\prime\prime} + ff^{\prime\prime} - f^{{\prime}{2}} - \gamma^{2} \left( {f^{\prime} - A} \right) + A^{2} = 0, $$

35 $$ \begin{aligned} & \left( {1 + 2\kappa \xi } \right)\left( {1 + \frac{4}{3}Rd} \right)\theta^{\prime\prime} + 2\kappa \left( {1 + \frac{4}{3}Rd} \right)\theta^{\prime} + \Pr {\text{Nb}}\left( {1 + 2\kappa \xi } \right)\left( {\theta^{\prime}\phi^{\prime}} \right) \\ & + \Pr \left( {f\theta^{\prime} - \delta_{1} f^{\prime} + Q\theta - \theta f^{\prime}} \right) + \Pr {\text{Nb}}\left( {1 + 2\kappa \xi } \right)\left( {\frac{{{\text{Nt}}}}{{{\text{Nb}}}}\theta^{{\prime}{2}} } \right) = 0, \\ \end{aligned} $$

36 $$ \left( {1 + 2\kappa \xi } \right)\left[ {\phi^{\prime\prime} + \frac{{{\text{Nt}}}}{{{\text{Nb}}}}\theta^{\prime\prime}} \right] + \Pr {\text{Le}}\left( {f\phi^{\prime} - \delta_{2} f^{\prime} - \phi f^{\prime}} \right) + 2\kappa \left( {\frac{{{\text{Nt}}}}{{{\text{Nb}}}}\theta^{\prime} + \phi^{\prime}} \right) - R_{c} \phi = 0, $$

**Table 4:** Comparison of SF with Ref.^57^.

$K$	$\beta$	$\Pr$	$0.5C_{f} {\text{Re}}_{x}^{{{1 \mathord{\left/ {\vphantom {1 2}} \right. \kern-\nulldelimiterspace} 2}}}$	
			Ref.^57^	Present results
0.4	0.1	0.1	− 2.3589	− 2.356854
0.5			− 2.7612	− 2.768647
0.6			− 3.1617	− 3.168475
0.1	1.1		− 2.3907	− 2.398325
	1.2		− 2.4330	− 2.434232
	1.3		− 2.4708	− 2.477993
	0.1	0.8	− 1.2276	− 1.225953
		1.0	− 1.2276	− 1.225953
		1.2	− 1.2276	− 1.225953

**Table 5:** Comparison of NN with Ref.^57^.

$K$	$\Pr$	$\delta_{1}$	${\text{Re}}_{x}^{{ - {1 \mathord{\left/ {\vphantom {1 2}} \right. \kern-\nulldelimiterspace} 2}}} {\text{Nu}}_{x}$	
			Ref.^57^	Present results
0.3	0.1	0.1	1.09438	1.094396
0.5			1.58186	1.581885
0.7			2.06024	2.060263
0.1	1.5		1.96336	1.963379
	1.7		2.05968	2.059691
	1.9		2.14648	2.146498
	0.1	0.2	0.36190	0.361918
		0.4	0.27146	0.271479
		0.6	0.18102	0.181038

**Table 6:** Comparison of SN with Ref.^57^.

$K$	$\delta_{2}$	Le	$\Pr$	${\text{Re}}_{x}^{{ - {1 \mathord{\left/ {\vphantom {1 2}} \right. \kern-\nulldelimiterspace} 2}}} {\text{Sh}}_{x}$	
				Ref.^57^	Present results
0.2	0.1	0.1		1.4913	1.495743
0.3				1.8466	1.847658
0.4				2.1845	2.187427
0.1	0.1			1.1297	1.128074
	0.2			1.0858	1.089863
	0.3			1.0420	1.046963
	0.1	0.4		1.1546	1.153520
		0.5		1.1627	1.169053
		0.6		1.1706	1.179063
		0.1	1.3	1.4752	1.476325
			1.5	1.5020	1.507964
			1.7	1.5271	1.526790

with transformed boundary conditions:

37 $$ \left. {\begin{array}{*{20}l} {f\left( \xi \right) = 0,\,\,\,f^{\prime}\left( \xi \right) = 1,\,\,\,\theta \left( \xi \right) = 1 - \delta_{1} ,\,\,\,\phi \left( \xi \right) = 1 - \delta_{2} ,\,\,\,at\,\,\,\xi = 0,} \hfill \\ {f^{\prime}\left( \xi \right) \to A,\,\,\,\theta \left( \xi \right) \to 0,\,\,\,\phi \left( \xi \right) \to 0,\,\,\,as\,\,\,\xi \to \infty } \hfill \\ \end{array} } \right\}. $$

### Phase 5

Ignoring the mixed convection phenomena and considering all other assumptions defined in Refs.^38,56,57^ with gyrotactic microorganisms through a stratified stretching cylinder is presented in this phase. The generalized mathematical modeling is given in (8–11) with boundary conditions (12). Here, we are interested to study the behavior of swimming microorganisms in a Casson fluid flow and to study the variation in Casson fluid flow through a stratified medium due to bioconvection Peclet number, bioconvection Lewis number, and concentration difference parameter. Furthermore, to analyze the flow of both magnetized and non-magnetized Casson fluid in the presence and absence of stagnation point. The physical quantities of interests like SF, NN, and SN are compared with the previous studies and have found quite similar results by which we have verified our study. Furthermore, the DN is calculated in Table 7. Here we have found that the DN increases with $K$, Pe, Le and $\Omega$ while the opposite trend in found via $\delta_{3}$.

**Table 7:** Numerical values of DN via different embedded parameters.

$K$	Pe	Le	$\delta_{3}$	$\Omega$	${\text{Re}}_{x}^{{ - {1 \mathord{\left/ {\vphantom {1 2}} \right. \kern-\nulldelimiterspace} 2}}} Nn_{x}$
0.1	0.1	0.1			− 1.106136
0.2					− 1.061690
0.3					− 1.017457
0.1	0.1				− 1.106136
	0.2				− 1.077995
	0.3				− 1.049848
	0.1	0.1			− 1.106136
		0.2			− 1.088843
		0.3			− 1.071553
		0.1	0.1		− 1.106136
			0.2		− 1.109254
			0.3		− 1.112386
			0.1	0.1	− 1.106136
				0.2	− 1.101374
				0.3	− 1.096625

#### Graphical results

**Figure 2:**
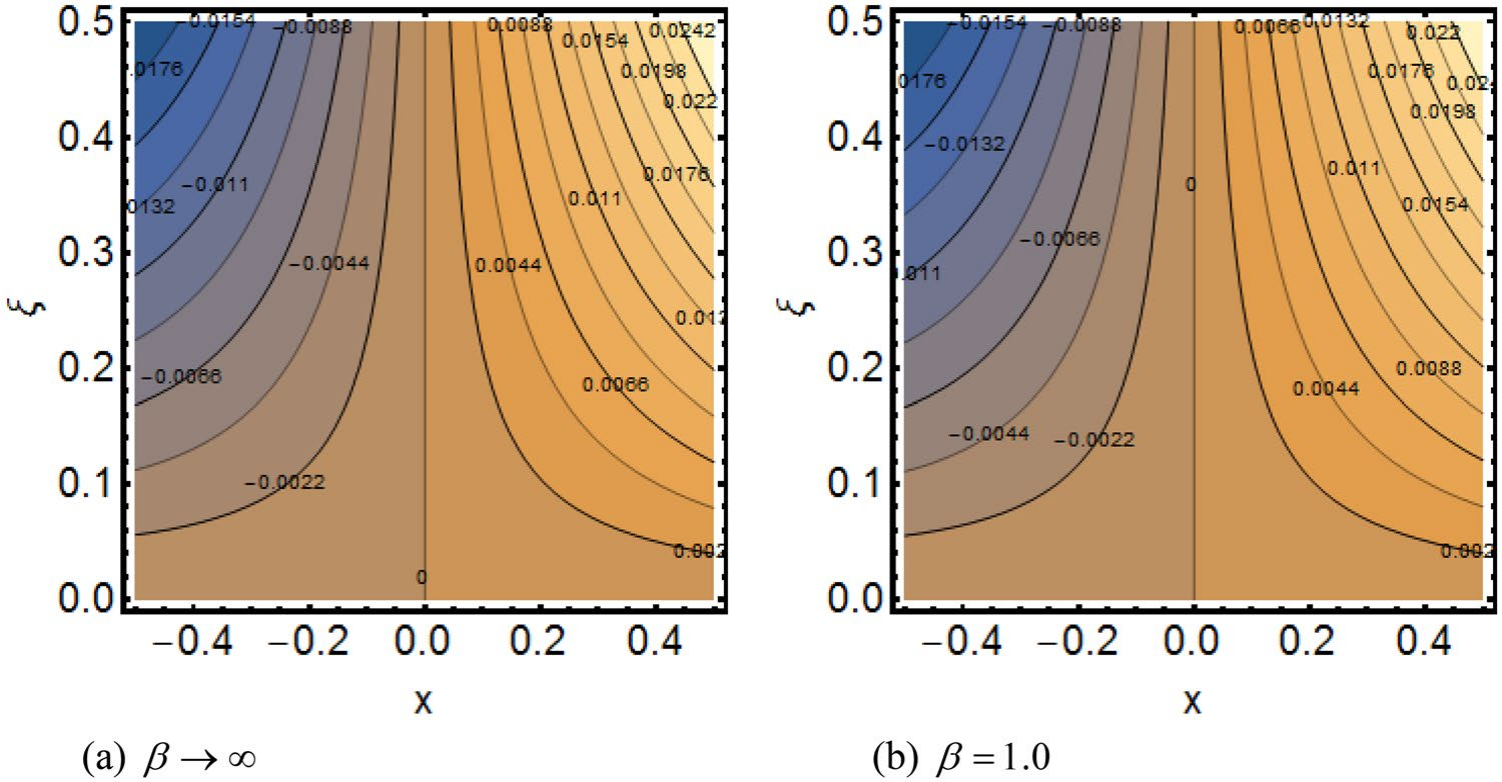
(**a**,**b**) Variations in the streamlines of Newtonian and non-Newtonian fluid velocity for magnetized stratified medium under the stagnation point.

**Figure 3:**
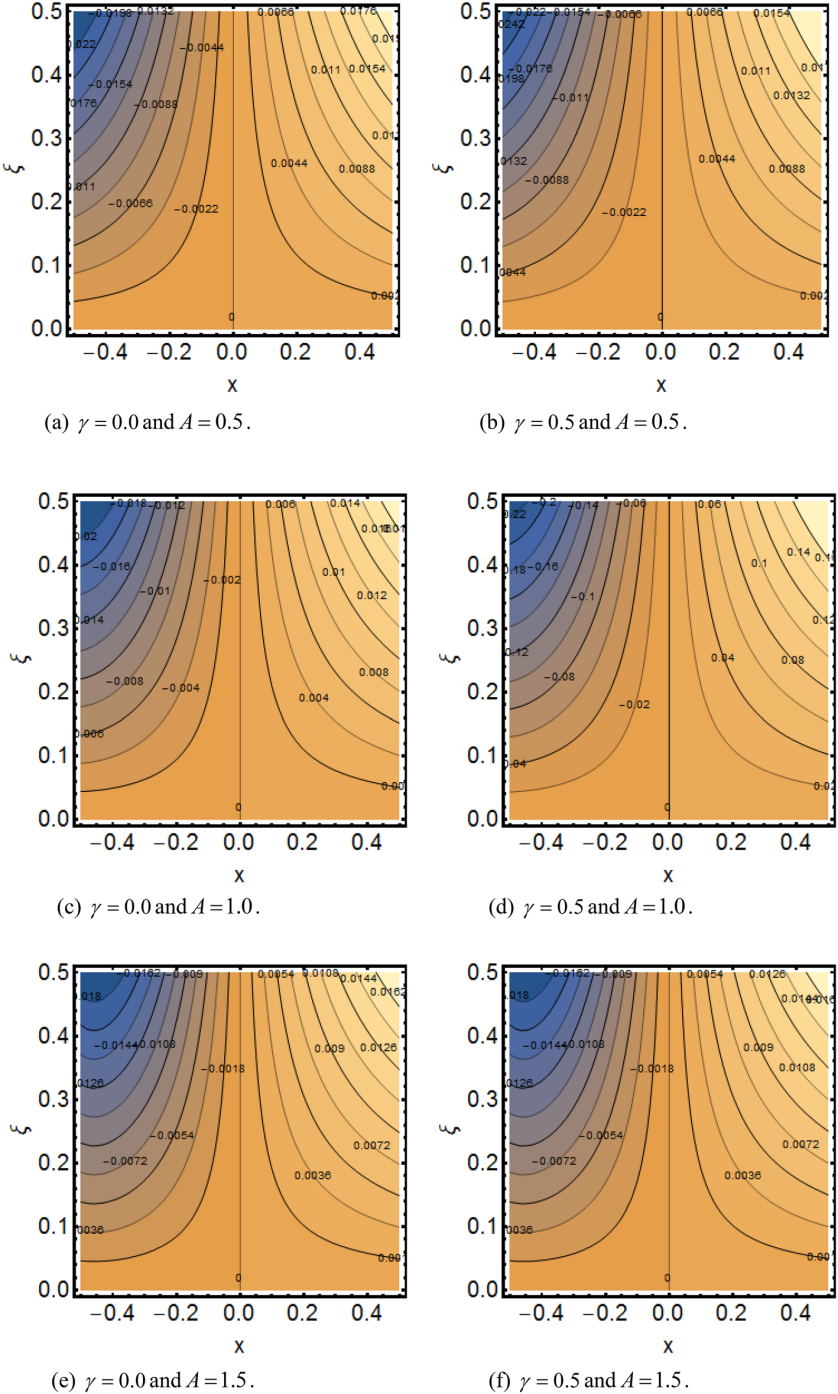
(**a**–**f**) Variations in the streamlines of Casson fluid velocity for both magnetized and non-magnetized stratified medium under the stagnation point.

**Figure 4:**
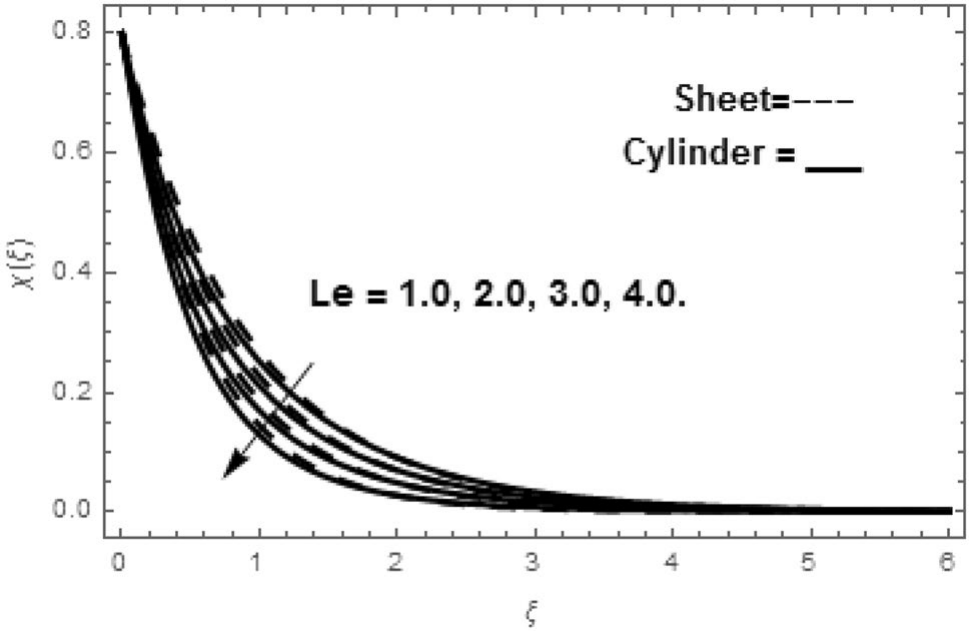
Variation in $\chi \left( \xi \right)$ via Le.

**Figure 5:**
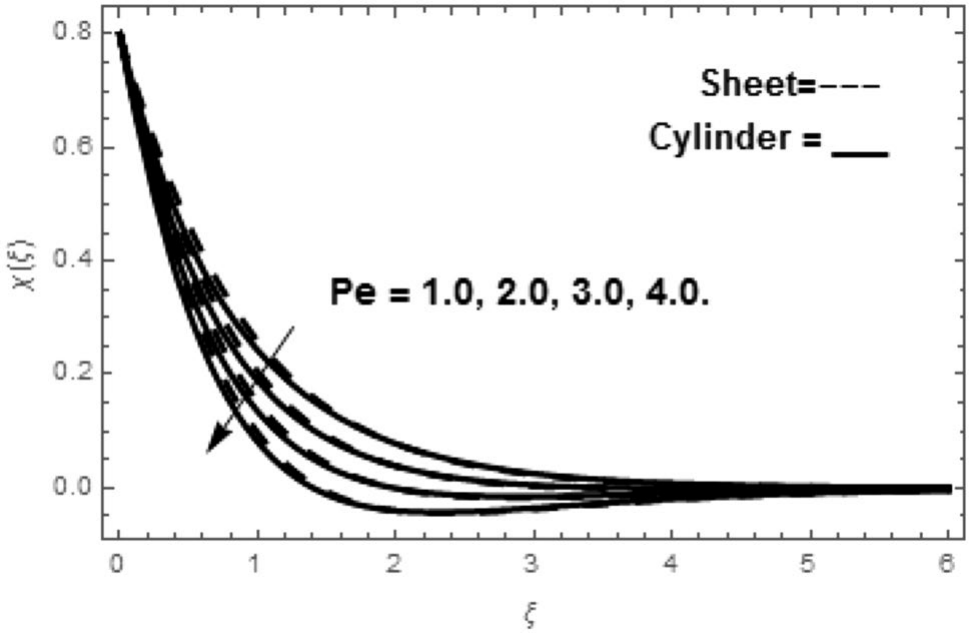
Variation in $\chi \left( \xi \right)$ via Pe.

**Figure 6:**
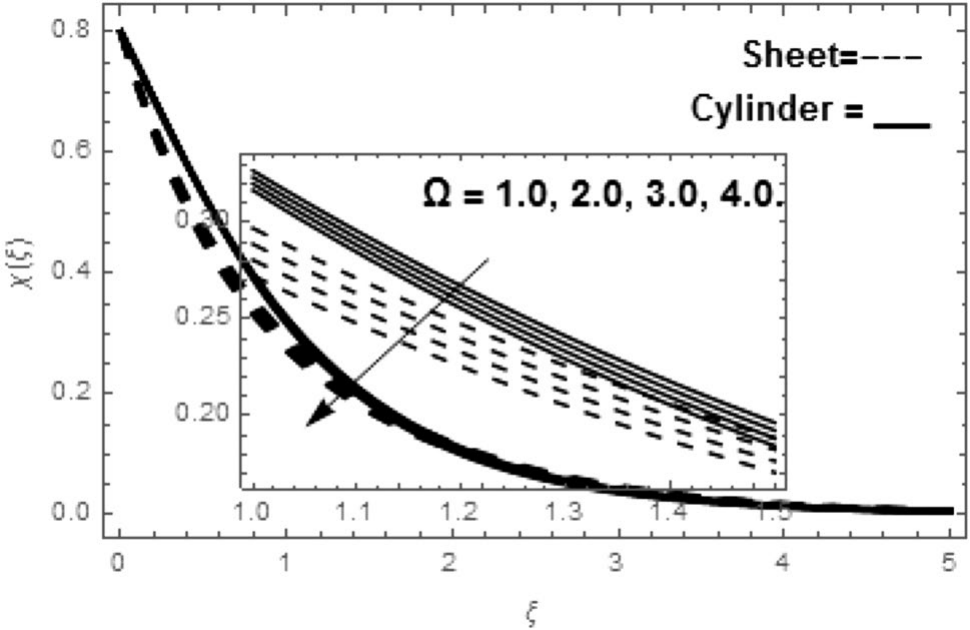
Variation in $\chi \left( \xi \right)$ via $\Omega$.

Figure 2a shows the variations in the streamlines of Newtonian fluid velocity for magnetized stratified medium under the stagnation point. Here, with the increasing Casson parameter (i.e.$\beta \to \infty$), we have observed that the streamlines become denser which results the increasing behavior in the fluid velocity, while on the other hand the fluid velocity reduces for the existence of Casson parameter (i.e.$\beta = 1.0$) as shown in Fig. 2b. Figure 3a–f show the variations in the streamlines of Casson fluid velocity for both magnetized and non-magnetized stratified medium under the region of stagnation point. Figure 3a,b are plotted for both non-magnetized and magnetized stratified medium when $A = 0.5$ respectively. We can see that the streamlines are wider for the case of magnetized Casson fluid as equated to non-magnetized fluid. Figure 3c,d are plotted for both non-magnetized and magnetized stratified medium when $A = 1.0$ respectively. Here, we observed that the streamlines are stifled for the case for the case of magnetized Casson fluid as equated to non-magnetized case. This conduct is due to the Lorentz force which always provides the contrasting force to the fluid flow. Figure 3e,f are plotted for both non-magnetized and magnetized stratified medium when $A = 1.5$ respectively. A similar impact of magnetic field is depicted as observed in Fig. 3a–d. Thus, we have concluded that the magnetic field plays a significant role in streamlines of Casson fluid flow. Figure 4 shows the influence of bioconvection Lewis number Le on motile density function. Here, we concluded that the higher values of Le reduce the motile density function. Figure 5 shows the influence of bioconvection Peclet Pe number on motile density function. The greater Peclet number reduces the diffusivity of microorganisms which conclude a reducing influence in motile density function. Thus, the greater Pe reduces the motile function of the microorganisms. Figure 6 shows the effect of microorganisms’ concentration difference parameter $\Omega$ on motile function. The increase in microorganisms’ concentration difference parameter heightens the microorganisms’ in the ambient liquid which consequently reduces the motile density function.

## Final comments

The mathematical model for both magnetized and non-magnetized Casson fluid containing gyrotactic microorganisms through a stretching cylinder is modeled under the effects of various parameter like stagnation point, Joule heating, heat absorption/generation, thermal stratification, mass stratification, motile stratification, nonlinear thermal radiation, magnetic field, and chemical reaction. We have studied the Casson fluid under different situations in order to analyze the flow behavior. The final comments are listed as:

- The increasing Casson parameter (i.e.$\beta \to \infty$) the streamlines become denser which results the increasing behavior in the fluid velocity, while on the other hand the fluid velocity reduces for the existence of Casson parameter (i.e.$\beta = 1.0$).
- The streamlines of stagnation point Casson fluid flow are highly wider for the case of magnetized fluid as equated to non-magnetized fluid.
- The motile function of microorganisms is reducing with higher values of bioconvection Lewis number, Peclet number, and microorganisms’ concentration difference parameter.
- The DN increases with higher values of curvature parameter, bioconvection Lewis number, Peclet number, and microorganisms’ concentration difference parameter while reduces with motile density stratification parameter.
